# Contributing to making the school a safe place for the child: School nurses’ perceptions of their assignment when caring for children having parents with serious physical illness

**DOI:** 10.1002/nop2.92

**Published:** 2017-09-07

**Authors:** Marie Golsäter, Karin Enskär, Susanne Knutsson

**Affiliations:** ^1^ CHILD ‐Research Group Department of Nursing Science School of Health and Welfare Jönköping University Jönköping Sweden; ^2^ Futurum Academy for Health and Care Region Jönköping County Jönköping Sweden

**Keywords:** children, content analysis, parental illness, school nurse, school situation

## Abstract

**Aim:**

To explore how school nurses perceive their assignment when caring for children having parents with serious physical illness.

**Design:**

An explorative inductive qualitative design.

**Method:**

The study is based on interviews with 16 school nurses. The interviews were subjected to qualitative content analysis.

**Results:**

The main category, “Contribute in making the school a safe place for the child”, reveals how the school nurses try to contribute to making the school a safe place for a child when his/her parent has a serious physical illness. They support children through individual support, as well as at an overall level in the school health team to make the school, as an organization, a safe place. Routines and collaboration to recognize the child when his/her parent has become ill is described as crucial to accomplishing this assignment.

## 1 INTRODUCTION

The school nursing assignment is to promote children's health to support the child in accomplishing the school situation (Socialstyrelsen 2014). Furthermore, the Swedish Health and Medical Act (Svensk Författningssamling (SFS), 1982) establish the obligation to consider children's need for information, advice and support when their parents have a serious illness or injury. Having a parent with a serious illness or injury, for example, cancer, is a condition that affects children's health and well‐being (Grabiak, Bender, & Puskar, 2007; Osborn, 2007; Socialstyrelsen, 2013; Visser, Huizinga, van der Graaf, Hoekstra, & Hoekstra‐Weebers, 2004) and thus can also affect the school situation (Hjern, Berg, Rostila, & Vinnerljung, 2013). When a parent becomes ill the family's everyday life changes (Davey, Tubbs, Kissil, & Niño, 2011; Huang, O'Connor, & Lee, 2014; Semple & McCaughan, 2013) and children have described how the illness rules the family's everyday life (Helseth & Ulfsæt, 2003).

Everything within a child and his/her environment affects the child's growth and development. Bronfenbrenner's Ecological Systems Theory (Bronfennbrenner, 1979) describes how a growing child is influenced by how the different environmental microsystems interact as part of a greater context. In the beginning, life revolves around the microsystem of the child and its family. As the child gets older his/her social network increases and he/she get new influences from other microsystem, for example, from school. These factors interact in any given situation for the child, such as when a parent is affected by illness (Bronfennbrenner, 1979).

As school is an important part of a child's everyday life, children express that the school staff need to be aware of their situation to provide support (Maynard, Patterson, McDonald, & Stevens, 2013). At the same time, children state that they only want to talk about their parent's illness with one or a few members of the school staff (Chalmers et al., 2000) and stress the importance of being treated normally (Huang et al., 2014; Kristjanson, Chalmers, & Woodgate, 2004). When the school manages to adjust the school situation to the child's needs, school attendance can be one aspect of helping the child handle the parent's illness (Chalmers et al., 2000; Kristjanson et al., 2004; Semple & McCance, 2010). Both academic support and emotional support from teachers and classmates are described as valuable (Bugge, Helseth, & Darbyshire, 2008; Chalmers et al., 2000; Huang et al., 2014), but telling about the parent's illness is difficult (Bugge et al., 2008; Maynard et al., 2013). Fasciano et al. (2007) show that school staff's greatest anxiety when facing families with a parent with cancer is how to communicate with the child and the parents. The experiences of the teachers reluctant of talking with the child about the parent's disease and how it affected the child's situation is described by children with parents suffering from multiple sclerosis (Bjorgvinsdottir & Halldorsdottir, 2014).

Based on the national health monitoring programme, all children in Sweden are invited to the school nurse for a health visit at least three times during their school years from 6 to 16 years of age. The health visit comprises a health dialogue about the child's health, lifestyle and everyday life. The school nurse is also one part of the school health team – consisting of a psychologist, counsellor, physician and special educator which has as the task of promoting each child's health and development towards his/her educational goals (Socialstyrelsen, 2014).

As the school is an important microsystem for a child's development, the interaction between the school and the child's family, the most important microsystem for a child, is crucial for the child's future health and well‐being (Bronfennbrenner, 1979). School nurses are part of the microsystem situated in schools, they are in a position to care for children, as well as collaborate with parents and other school staff to adjust the school situation for children having a parent with a serious illness (SFS, 1982, Socialstyrelsen 2014). When supporting children, whose parents suffer from ill mental health, school nurses describe the assignment as challenging and stress the need for collaboration with both parents and staff at school (Hammarlund, Falk, Lind, & Thorstensson, 2015). However, to the best of our knowledge research regarding school nurses work when caring for children having parents with serious physical illness is limited. Therefore, to develop the support in school for a child when his/her parent has a serious physical illness there is a need to explore school nurses’ perceptions of their role in this assignment.

### 1.1 Aim

The aim was to explore how school nurses perceive their assignment when caring for children having parents with serious physical illness.

## 2 METHODS

This study was based on an explorative inductive qualitative design (Polit & Beck, 2012) where 16 school nurses were interviewed.

### 2.1 Participants

To receive a purposeful sample the head of the school nurses were informed about the study and contacted school nurses with experience of supporting children having a parent with a serious physical illness (e.g. cancer, traffic‐accidents or neurological diseases), further they should have a specialist nursing education and more than 2 years’ experience working as a school nurse. The interviews were performed in one Swedish county and the participating school nurses were all female, worked with children aged 6–16 years in different socioeconomic areas.

### 2.2 Data collection

The 16 individual interviews were performed at the school nurses’ workplaces. Four nursing students at an advanced level conducted the interviews under supervision of the last author (SK) in the frame of a larger research project regarding children as relatives. Each student made a pilot interview on which the last author (SK) gave feedback and suggestions for improvements. The pilot interviews are included in the material. The interviews were based on one open question: “Can you tell me how you perceive your assignment when caring for children having a parent with a serious physical illness?” To enable a reflective dialogue, probing questions like “How do you mean” and “Could you explain further” were used (Kvale & Brinkman, 2014). The interviews were performed between September 2014 and February 2015; audio recorded and lasted 20–55 min.

### 2.3 Data analysis

The verbatim transcribed data were analysed according to inductive content analysis as described by Elo and Kyngas (2008). One of the researchers participating in the analysis had experiences of working as a school nurse and all of them were working in a research project about children as relatives. Each interview was read several times by the authors independently to capture essential features and obtain a sense of the content. In the next step, statements reflecting the nurses’ perceptions were described as codes. The authors then compared and discussed their written codes to reach agreement and grouped the codes into sub‐categories. By going back and forth among the codes and sub‐categories, four generic categories were formed based on their similarities and differences. The generic categories where then further abstracted in a main category describing how the school nurses’ perceived their assignment: “Contribute in making the school a safe place for the child” (Table 1).

**Table 1:** Overview of the generic categories and the main category

Generic categories	Main category
To recognize the child	Contribute in making the school a safe place for the child
To involve the parents	
To provide care tailored to the child's own terms	
To collaborate with other professionals	

### 2.4 Ethical consideration

The study was performed according to the Swedish law stating that ethical approval is not needed when interviewing healthcare professionals about work‐related questions (SFS, 2003) and according to the Declaration of Helsinki (World Medical Association, 2009). The head of the school nurses were informed about the study and signed an informed consent. Before the interviews the participants received both oral and written information about the study, confidentiality, how the data would be handled and that they could withdraw participation at any time and signed an informed consent.

## 3 FINDINGS

The main category: “Contribute in making the school a safe place for the child” reveals how the school nurses perceive their assignment in caring for a child when his/her parent had a serious physical illness. Through their experiences and knowledge about children, the nurses describe that they, in collaboration with parents and school staff, can contribute to create a school environment that best supports a good education and well‐being for the child. At an overall level, the school nurses contribute to making the school, as an organization, a safe place for the child by collaborating with and supporting the school health team and the child's teacher based on their knowledge as a nurse. At an individual level, the nurses describe how they in collaboration with the parents contribute in making the school a safe place for the child both physically (providing the child the opportunity to come to their office) and psychologically (being emotional with the child). Routines and collaboration to recognize the child when his/her parent has become ill is described as crucial to accomplishing this assignment.

### 3.1 To recognize the child

The nurses argue that one main problem when supporting children as relatives is the lack of structured routines about how they learn that a parent of a child at their school is severely ill. It could often happen by coincidence and long after the parent had been diagnosed, that they learned of the child's situation. This hampered their ability to support the child. Sometimes the parents themselves contact the nurse either through direct contact with the nurse or with the teachers who had had contact with the parents:

You don't find out about them so very easily because there aren't so many parents who tell…the school nurse isn't the first person they contact

In conjunction with a regular health visit a child will sometimes express concern about his/her parent's situation and through this the nurse learns of the parent's illness. In some cases, the school nurses get to know about the situation when a child visits them for vague symptoms and the nurses try to figure out what the problem is by asking questions about the child's everyday life the child starts to talk about the parent's situation.

As confidential rules restrict the contact between various healthcare providers, the school nurses state that there are no obvious pathways for information transfer between the nurses caring for the parent and the child's school nurse. However, the school nurses describe that often both parents and teachers believe the school nurse works in conjunction with nurses in other parts of the healthcare system and thus always gets information when a child's parent becomes ill. The school nurses argue for communication between the school nurse and the nurse caring for the parent as one way to the strengthen support to both children and their families in the different parts of the healthcare system. The school nurses describe that nurses working with adult patients might not be aware of how the school nurses work and how they can take part in supporting children. The school nurses suggest that nurses caring for the parent consider asking the parent to contact the school nurse to care for and support the child. The school nurses describe that they do not need specific information about prognoses or treatments, but only general information about the parent's condition and what kind of support the child is receiving from the nurses caring for the parent, to provide additional support based on the child's needs:

It never happens that there comes a call or a referral or something from the hospital…it actually hinges on the parent notifying…or when I had a health dialogue in fourth grade and the child says my dad has cancer…

To obtain a structure for the communication between school nurses and nurses caring for parents, the school nurses describe that it might be helped by a form containing questions about contact with other Health Care professionals (HCP), for example, the school nurse, to facilitate their work with adult patients who have children.

### 3.2 To involve the parents

The school nurses describe that a prerequisite for being able to care for the child is that the support is accomplished in agreement with the parents. This means that the first thing that should be done is to obtain the parents’ permission to talk to the child and to provide support:

The teacher can contact me if they notice a child isn't feeling well when a parent is ill, but then you can't just ask the child to come; you have to call the parents first and see if they want you to meet with the child

However, the agreement may differ depending on the child's age. The nurses describe that, based on their knowledge of supporting children and families with different needs and in different life situations; they can also facilitate for the parent to understand how this new situation affects the child and through this support the child and the family. The fact that someone is caring for their child is something the nurses perceive to be valued by parents. Through their support to the child, the school nurses adjust to how the family has chosen to handle and talk about the parent's illness. Having continuous contact with the parents via email or telephone as well as keeping a form of diary together with the child are described as different ways to maintain this contact.

### 3.3 To provide care tailored to the child's own terms

Through their education and professional experience of encountering children, the school nurses describe having the ability to provide individual care to a child when his/her parent is ill.

In their work at the school, the nurses describe that they can, in a natural way, easily contact the child to let he/she know they are aware of the parent's situation. When the nurses encounter these children, their strategies are to try to exude confidence and trust by showing that they are listening and that they understand the child's situation and are there for him/her. By being calm and confident they try to assure the child that it is all right to talk and that supporting children who have a parent with serious illness is a natural part of school nurse's work. The nurses describe that, through showing that it is normal to talk to the school nurse, they try to de‐dramatize and normalize the situation to show that it is normal for a parent's illness to affect the child.

The nurses describe that, in the encounter, they try to get a picture of the child's situation and need for support through probing questions and talking about the child's everyday life. In some cases, they use information from an earlier health visit and, based on this, let the child talk about the situation today. The nurses describe that letting the child describe everyday life allows them to facilitate for him/her to understand how the parent's illness is affecting him/her, which the child may not have previously reflected on or been aware of.

The school nurses describe that children have a need to feel secure and to know there is someone they can turn to when necessary; the school nurses describe trying to be that person. Children desire a school situation that is as normal as possible and thus might not want to discuss their situation with a teacher they see every day. Instead, according to the nurses, it could be easier to turn to the nurse at her office if something comes up during the school day that is hard to handle.

The nurses describe that, if the child expresses a desire for continued contact they try to work out how to best tailor the support. The nurses state that it is important to let the child decide what to talk about. Sometimes, the nurse could meet with the child several times without talking about the parent's illness or how it is affecting the child, instead letting him/her talk about more harmless subjects:

I have a boy who I meet with every other week and follow and check the schedule and just talk in general…it's not always that you sit there and talk about the hard things; sometimes maybe we just play Noughts and Crosses and just “are”.

The nurses interpret this as a way of letting the child determine whether the school nurse is trustworthy. In the encounters, they try to be encouraging, focusing on everyday activities, for example, continuing with leisure activities. To be able to do this, the nurses argue that they should not be too keen; instead providing the child with space and helping him/her feel at ease in the situation. Through the conversation, they try to help the child put words to their feelings and thoughts and listen to the child's story without interrupting or giving advice. One form of support they describe involves helping the child find answers to his/her questions about the parent's illness and treatment. The nurses could answer some of the questions based on their own knowledge, while some were answered by looking at web pages with information for children together with the child. Helping the child formulate questions to the HCP caring for the parent is also described as a way of supporting the child.

### 3.4 To collaborate with other professionals

To provide the best possible support for the child, the school nurses argue for the importance of collaborating with others both in the school and in other parts of the healthcare system. By cooperating with others in the school, such as teachers and school counsellors, the nurses try to get an overview of the child's school situation, how he/she is managing the school work and how he/she interacts with school mates. Through this overview the nurse can decide, together with the school health team and the child's form teacher, how to arrange the school situation to support the child in having the best possibilities to manage the education and social relations:

Then I report back, after the child's consent and the parents’ consent, to the school health team so we know what's going on…

The most important task described by the nurses is ensuring that there is clear agreement on who has the main responsibility to maintain contact with the child and the parents and keeping in mind the child's situation to offer support at an early stage.

In most cases it is the form teacher who has this role, as he/she is the one who has daily contact with the child. Keeping in mind the child's situation, incorporates an overview of his/her school results and attendance, as well as the situation with his/her school mates. By collaborating in the school health team, the different professionals, based on their different knowledge, can together support the child.

Through keeping in mind the child's situation and collaborating in the school health team, worries about how he/she is acting could be handled at an early stage and unnecessary investigations could be avoided. For example, support could be provided through more individual educational help from teachers or additional contact with the school counsellor or the school nurse. Another way to give support through collaboration, described by the nurses, is to refer the child and the family to other parts of the healthcare services if they have needs exceeding what the school health team can provide.

## 4 DISCUSSION

The school nurses described how they try to contribute in making the school a safe place for the child, to support the child in handling the situation of illness in the family. Alliance with the child, the child's parents, as well as collaboration in the school and other HCP are crucial to perform this. To accomplishing this assignment, the nurses describe the need of routines and collaboration to recognize the child when his/her parent has become ill.

Bronfenbrenner's model describes the different microsystems interactions at the meso‐level as central for the child's health and well‐being (Bronfennbrenner, 1979). This study incorporates primarily the two microsystems, the family and the school and the cooperation between these at the meso‐level but incorporates also the Health Care Services caring for the parent.

The school nurses in the present study describe themselves as competent and prepared to care for children whose parent has a serious illness. Based on children's wish to talk to a HCP about their parent's illness (Karlsson, Andersson, & Ahlström, 2013; Patterson, Pearce, & Slawitschka, 2011) and to be treated normally at the school (Huang et al., 2014; Kristjanson et al., 2004) this shows that school nurses can support children based on their professional knowledge and experience of encountering children. Furthermore, school nurses have a natural way of contacting children as they are situated in the school and as part of the school health team they have an impact on the child's school situation and can thus tailor it according to the child's own wishes (Socialstyrelsen, 2014).

Having an HCP to easily contact on their own if needed is described as useful by the children in Kristjanson et al. (2004) which is in line with what the school nurse described trying to achieve by encountering the child and telling they are aware of his/her situation. This encounter with the child can also be considered as one way to allow the child access to an HCP and age‐appropriate information, which children has described a lack of (Kennedy & Lloyd‐Williams, 2009; Kristjanson et al., 2004). Receiving information about the parent's treatment and its side effects is described as crucial for children's possibility to understand the parent's situation and why he/she has acted in an unfamiliar way (Kristjanson et al., 2004; Maynard et al., 2013 and how this may affect the family as a microsystem (Bronfennbrenner, 1979).

As the school is one of the important microsystems of a child's everyday life (Bronfennbrenner, 1979), it is important that he/she can feel comforted there. Circumstances in the family, such as a parent's illness, affect the school situation as the two environmental systems, family and school; interact with each other in the situation for the child in agreement with Bronfenbrenner's Ecological Systems Theory (Bronfennbrenner, 1979). Thus, it is important that the interaction, at the meso‐level, between the child's family and school is well established to promote the child's health and well‐being.

The school nurses describe that, when they encounter these children they try to get a picture of their situation to see how they can tailor the support according to the child's needs. Adolescents describe support tailored to their own needs as the most useful kind of support from an HCP when a parent is ill (Kristjanson et al., 2004) and stress the importance of the HCP being thoughtful and keeping the conversation confidential (Kennedy & Lloyd‐Williams, 2009). Having the support from the school nurse tailored to one's own needs is also described by children encountering school nurses for other needs where a trustful relationship is described as crucial to feel secure (Golsater, Sidenvall, Lingfors, & Enskar, 2010). Children taking part in a support programme for children with a parent with cancer describe first feeling a bit frightened to talk on their own with professionals, as they did not know them (Bugge et al., 2008). This is in line with the experiences of children being offered support in conjunction with a parent's death, who declined the support as they did not want to talk to a stranger (Karlsson et al., 2013). Through her work at the school incorporating regular health visits, the school nurse is known to the children and thus might be easier for the child to talk to, compared with an unknown HCP at the hospital. However, children also state a wish to meet the HCP taking care of their parent and to receive information about the illness and the treatment (Bugge et al., 2008), which also argues for more communication between the nurses caring for the parent and the school nurse to fulfil children's different needs regarding information, advice and support, which is stated in the Swedish Health and Medical Service Act (SFS, 1982).

The present study reveals a lack of routines in the communication between the school and the unit caring for the parent. This is in line with the results from Chalmers et al. (2000) whereby children from the age of 12 describe a lack of clear routines and collaboration between the school and the unit caring for the parent. The need to further develop routines in healthcare services to care for children as relatives is earlier shown (Knutsson, Enskär, Andersson‐Gäre, & Golsäter, 2017) and the variations of how nurses working with adult patients perceive their role in caring for children as relatives (Golsäter, Henricson, Enskär, & Knutsson, 2016) argue for the need to improve the communication to be able to support the child.

The results from the present study also reveal that parents may not always be aware of the school nurse's possibilities to support the child, as they do not initiate contact with the school nurse. Parents have requested for more collaboration with their children's school nurse and knowledge about the nurse's assignment (Maenpaa & Astedt‐Kurki, 2008a; Mäenpää, Paavilainen, & Åstedt‐Kurki, 2013; Read, Small, Donaher, Gilsanz, & Sheetz,^2009^) to promote the child's health. However, discrepancies between nurses’ and parents’ experiences of collaboration between these two parties are shown in earlier studies (Maenpaa & Astedt‐Kurki, 2008a, 2008b; Mäenpää et al., 2013). Insufficient information from and contact with school nurses are described by parents, while school nurses describe that they are the ones who have to take the initiative in contact with parents (Maenpaa & Astedt‐Kurki, 2008a, 2008b). These somehow contradictory experiences can be one reason why parents wait to contact the school nurse when one of them becomes ill; they may not be aware of the support school nurses can provide. Earlier studies have also shown that parents, in their ambition to protect their child, choose not to inform him/her to any greater extent about the illness; this is described as a balancing act between seeing to the child's need to be involved and at the same time maintaining his/her security (Billhult & Segesten, 2003; Helseth & Ulfsæt, 2005; Semple & McCance, 2010).This could be another reason for parents choosing not to contact the school nurse. To overcome this, Mäenpää et al. (2013) argue for a closer family–school nurse partnership. In a closer partnership, both school nurse and parent can be aware of how the family and the school situation interact and by that facilitate the interaction at meso‐level and through this promote the child's health in line with Bronfenbrenner's Ecological Systems Theory (Bronfennbrenner, 1979).

At an overall level, the school nurses describe collaborating with the school health team and the child's teacher to make the school a safe place for the child. Based on children's desire to be treated normally (Huang et al., 2014; Kristjanson et al., 2004) and to not have to talk about their parent's illness with everyone at school, the school nurses in this study try to be someone the child can come to when needed but do not have to face every day like their teachers. The nurses also describe that through their work in the school health team, they try to support the teachers who have the closest contact with the child; this way of working is also described in Fasciano et al. (2007). Furthermore, the nurses state that the members of the school health team with their different professions and knowledge are crucial in enabling the school situation to be that supportive place, both emotional and academic, that children desire (Chalmers et al., 2000; Kristjanson et al., 2004; Semple & McCance, 2010). One way to further promote a child's health during his/her parent's illness could be to work with family sessions, as described by Clausson and Berg (2008). This could be one way for school nurses to develop the support in conjunction with Mäenpää et al. (2013) description of Family–School nurse partnership, with the goal of being there for the child and the family.

### 4.1 Methodological considerations

The participants in this study consisted of a purposeful sampling (Polit & Beck, 2012) of school nurses from one county in Sweden. Based on the aim of the study, they all had experience of working as school nurses at different schools and with children of different ages, which contributes to the substantial findings of the study (Polit & Beck, 2012). However, a limitation of the study is that all the participants were from just one county in Sweden and this has to be taken under consideration according to transferability of the findings. Another possible limitation is that the interviewers were not trained interviewers. To handle this, the interviews were performed under supervision from an experienced researcher. Also, the researchers who performed the analysis were not present during the interviews which can be considered as a limitation. To ensure trustworthiness throughout the analysis process the analysis was performed in ongoing discussion between the three authors to reveal alternative interpretations of the data. All authors have previous experience with content analysis (Elo et al., 2014). Quotations from the interviews are presented to support the analytic results (Polit & Beck, 2012).

## 5 CONCLUSION

The school nurses in the present study describe that they, as they are situated in the school and through their experience and knowledge about children, can support children in handling the situation of illness in the family. Together with parents and school staff, the school nurse can act as a facilitator to create a school environment that best supports a good education and everyday life for a child whose parents are suffering from serious physical illness. The results from this study argue for closer collaboration between parents, nurses caring for parents, school nurses and teachers to take advantage of the possibility to develop support in the school for children whose parent has a serious physical illness. The school nurse can be a part in facilitating the interaction between two important microsystems in children's everyday life; the family and the school, to promote the child's health and well‐being. To accomplish this assignment, a prerequisite is to develop routines that facilitate for the school nurse to recognize the child.

## CONFLICT OF INTEREST

No conflict of interest has been declared by the author(s)’.

## AUTHOR CONTRIBUTION

Marie Golsäter, Karin Enskär and Susanne Knutsson designed the study and performed the data analysis. Susanne Knutsson performed the data collection, Marie Golsäter and Susanne Knutsson drafted the manuscript and all authors have accepted the final version of the manuscript.

All authors have agreed on the final version of the paper and meet at least one of the following criteria (based on those recommended by the ICMJE):

- substantial contributions to conception and design, acquisition of data or analysis and interpretation of data
- drafting the article or revising it critically for important intellectual content.

## References

[nop292-bib-0001] Billhult, A. , & Segesten, K. (2003). Strength of motherhood: Nonrecurrent breast cancer as experienced by mothers with dependent children. Scandinavian Journal of Caring Sciences, 17, 122–128. 12753512 10.1046/j.1471-6712.2003.00219.x

[nop292-bib-0002] Bjorgvinsdottir, K. , & Halldorsdottir, S. (2014). Silent, invisible and unacknowledged: Experiences of young caregivers of single parents diagnosed with multiple sclerosis. Scandinavian Journal of Caring Sciences, 28, 38–48. 23550661 10.1111/scs.12030

[nop292-bib-0003] Bronfennbrenner, U. (1979). The ecology of human development – experiments by nature and design. Cambridge, Massachusetts: Harvard University Press.

[nop292-bib-0004] Bugge, K. E. , Helseth, S. , & Darbyshire, P. (2008). Children's experiences of participation in a family support program when their parent has incurable cancer. Cancer Nursing, 31, 426–434. 18987509 10.1097/01.NCC.0000339250.83571.b0

[nop292-bib-0005] Chalmers, K. I. , Kristjanson, L. J. , Woodgate, R. , Taylor‐Brown, J. , Nelson, F. , Ramserran, S. , & Dudgeon, D. (2000). Perceptions of the role of the school in providing information and support to adolescent children of women with breast cancer. Journal of Advanced Nursing, 31, 1430–1438. 10849156 10.1046/j.1365-2648.2000.01449.x

[nop292-bib-0006] Clausson, E. , & Berg, A. (2008). Family intervention sessions one useful way to improve schoolchildren's mental health. Journal of Family Nursing, 14, 289–313. 18780887 10.1177/1074840708322758

[nop292-bib-0007] Davey, M. P. , Tubbs, C. Y. , Kissil, K. , & Niño, A. (2011). “We are survivors too”: African‐american youths’ experiences of coping with parental breast cancer. Psycho‐Oncology, 20, 77–87. 20198717 10.1002/pon.1712

[nop292-bib-0008] Elo, S. , Kääriäinen, M. , Kanste, O. , Pölkki, T. , Utriainen, K. , & Kyngäs, H. (2014). Qualitative content analysis. SAGE Open, 4, 2158244014522633.

[nop292-bib-0009] Elo, S. , & Kyngas, H. (2008). The qualitative content analysis process. Journal of Advanced Nursing, 62, 107–115. 18352969 10.1111/j.1365-2648.2007.04569.x

[nop292-bib-0010] Fasciano, K. M. , Berman, H. , Moore, C. , DeFrino, B. , Jameson, R. , Kennedy, V. , & Golant, M. (2007). When a parent has cancer: A community based program for school personnel. Psycho‐Oncology, 16, 158–167. 17273985 10.1002/pon.1148

[nop292-bib-0011] Golsäter, M. , Henricson, M. , Enskär, K. , & Knutsson, S. (2016). Are children as relatives our responsibility? –How nurses perceive their role in caring for children as relatives of seriously ill patients. European Journal of Oncology Nursing, 25, 33–39. 27865250 10.1016/j.ejon.2016.09.005

[nop292-bib-0012] Golsater, M. , Sidenvall, B. , Lingfors, H. , & Enskar, K. (2010). Pupils’ perspectives on preventive health dialogues. British Journal of School Nursing, 5, 26–33.

[nop292-bib-0013] Grabiak, B. R. , Bender, C. M. , & Puskar, K. R. (2007). The impact of parental cancer on the adolescent: An analysis of the literature. Psycho‐Oncology, 16, 127–137. 16998950 10.1002/pon.1083

[nop292-bib-0014] Hammarlund, K. , Falk, J. , Lind, J. , & Thorstensson, S. (2015). Meeting and supporting students who have parents with mental ill‐health. British Journal of School Nursing, 10, 182–187.

[nop292-bib-0015] Helseth, S. , & Ulfsæt, N. (2003). Having a parent with cancer: Coping and quality of life of children during serious illness in the family. Cancer Nursing, 26, 355–362. 14710796 10.1097/00002820-200310000-00003

[nop292-bib-0016] Helseth, S. , & Ulfsæt, N. (2005). Parenting experiences during cancer. Journal of Advanced Nursing, 52, 38–46. 16149979 10.1111/j.1365-2648.2005.03562.x

[nop292-bib-0017] Hjern, A. , Berg, L. , Rostila, M. , & Vinnerljung, B. (2013). Barn som anhöriga ‐ hur går det i skolan.(Children as relatives – how is it going in school). Kalmar: Nationellt Kompetenscentrum för anhöriga.

[nop292-bib-0018] Huang, X. , O'Connor, M. , & Lee, S. (2014). School‐aged and adolescent children's experience when a parent has non‐terminal cancer: A systematic review and meta‐synthesis of qualitative studies. Psycho‐Oncology, 23, 493–506. 24323875 10.1002/pon.3457

[nop292-bib-0019] Karlsson, E. , Andersson, K. , & Ahlström, B. H. (2013). Loneliness despite the presence of others –Adolescents’ experiences of having a parent who becomes ill with cancer. European Journal of Oncology Nursing, 17, 697–703. 24183584 10.1016/j.ejon.2013.09.005

[nop292-bib-0020] Kennedy, V. L. , & Lloyd‐Williams, M. (2009). Information and communication when a parent has advanced cancer. Journal of Affective Disorders, 114, 149–155. 18684513 10.1016/j.jad.2008.06.022

[nop292-bib-0021] Knutsson, S. , Enskär, K. , Andersson‐Gäre, B. , & Golsäter, M. (2017). Children as relatives to a sick parent: Healthcare professionals’ approaches. Nordic Journal of Nursing Research, 37, 61–69.

[nop292-bib-0022] Kristjanson, L. J. , Chalmers, K. I. , & Woodgate, R. (2004). Information and support needs of adolescent children of women with breast cancer. Oncology Nursing Forum, 31, 11–119. 14722595 10.1188/04.ONF.111-119

[nop292-bib-0023] Kvale, S. , & Brinkman, S. (2014) Den kvalitativa forskningsintervjun (The qualitative research interview). 3rd ed. Lund: Studentlitteratur.

[nop292-bib-0024] Maenpaa, T. , & Astedt‐Kurki, P. (2008a). Cooperation between parents and school nurses in primary schools: Parents’ perceptions. Scandinavian Journal of Caring Sciences, 22, 86–92. 18269427 10.1111/j.1471-6712.2007.00527.x

[nop292-bib-0025] Maenpaa, T. , & Astedt‐Kurki, P. (2008b). Cooperation between Finnish primary school nurses and pupils’ parents. International Nursing Review, 55, 219–226. 18477107 10.1111/j.1466-7657.2007.00608.x

[nop292-bib-0026] Mäenpää, T. , Paavilainen, E. , & Åstedt‐Kurki, P. (2013). Family–school nurse partnership in primary school health care. Scandinavian Journal of Caring Sciences, 27, 195–202. 22616916 10.1111/j.1471-6712.2012.01014.x

[nop292-bib-0027] Maynard, A. , Patterson, P. , McDonald, F. E. , & Stevens, G. (2013). What is helpful to adolescents who have a parent diagnosed with cancer? Journal of Psychosocial Oncology, 31, 675–697. 24175902 10.1080/07347332.2013.835021

[nop292-bib-0028] Osborn, T. (2007). The psychosocial impact of parental cancer on children and adolescents: A systematic review. Psycho‐Oncology, 16, 101–126. 17273987 10.1002/pon.1113

[nop292-bib-0029] Patterson, P. , Pearce, A. , & Slawitschka, E. (2011). The initial development of an instrument to assess the psychosocial needs and unmet needs of young people who have a parent with cancer: Piloting the offspring cancer needs instrument (OCNI). Supportive Care in Cancer, 19, 1165–1174. 20574665 10.1007/s00520-010-0933-7

[nop292-bib-0030] Polit, D. , & Beck, C. (2012). Nursing research, 9th ed Philadelphia: Lippincott Williams & Wilkins.

[nop292-bib-0031] Read, M. , Small, P. , Donaher, K. , Gilsanz, P. , & Sheetz, A. (2009). Evaluating parent satisfaction of school nursing services. Journal of School Nursing, 25, 205–213. 19363109 10.1177/1059840509334441

[nop292-bib-0032] Semple, C. J. , & McCance, T. (2010). Parents’ experience of cancer who have young children: A literature review. Cancer Nursing, 33, 110–118. 20142737 10.1097/NCC.0b013e3181c024bb

[nop292-bib-0033] Semple, C. J. , & McCaughan, E. (2013). Family life when a parent is diagnosed with cancer: Impact of a psychosocial intervention for young children. European Journal of Cancer Care, 22, 219–231. 23231498 10.1111/ecc.12018

[nop292-bib-0034] Socialstyrelsen (2013). Barns och ungas hälsa, vård och omsorg (Children and Adolescents Health and Health care). Stockholm: Socialstyrelsen 2013 Contract No: 2013‐3‐15

[nop292-bib-0035] Socialstyrelsen (2014). Vägledning för Elevhälsan (Guidelines for School Health Care). Stockholm: Socialstyrelsen.

[nop292-bib-0036] Svensk Författningssamling (SFS) (2003:460). Lag om etikprövning av forskning som avser människor (Act on Ethical Scrutiny of Research Involving Human beings).

[nop292-bib-0037] Svensk Författningssamling (SFS) (1982:763 § 2 g). Hälso‐ och sjukvårdslagen (Health and MedicalServices Act)Stockholm, Sweden: Ministry of Health and Social Affairs.

[nop292-bib-0038] Visser, A. , Huizinga, G. A. , van der Graaf, W. T. , Hoekstra, H. J. , & Hoekstra‐Weebers, J. E. (2004). The impact of parental cancer on children and the family: A review of the literature. Cancer Treatment Review, 30, 683–694. 10.1016/j.ctrv.2004.06.001 15541578

[nop292-bib-0039] World Medical Association (2009). Medical ethics manual. 2nd ed, Available from http://www.wma.net/en/30publications/30ethicsmanual/pdf/ethics_manual_en.pdf (2009, acessed 5 November 2015).Association WM. Medical ethics Manual. 2009.

